# Initial Experience with the New DERIVO^®^ Mini Embolisation Device for the Treatment of Intracranial Aneurysms

**DOI:** 10.3390/brainsci14090911

**Published:** 2024-09-10

**Authors:** Sinan Balci, Ferdi Çay, Aycan Uysal, Anil Arat

**Affiliations:** 1 Department of Radiology, Hacettepe University, 06230 Ankara, Turkey; snnbalci@gmail.com (S.B.); drferdicay@gmail.com (F.Ç.); aycanuysal@hotmail.com (A.U.); 2 Department of Neurosurgery, Yale University, New Haven, CT 06510, USA

**Keywords:** small profile, flow diversion, intracranial aneurysm, embolization, endovascular treatment

## Abstract

The aim of this study is to present the outcomes of cerebral aneurysm treatment with the DERIVO^®^ mini Embolisation Device (DMD), which is compatible with microcatheters with 0.021-inch inner diameters. Consecutive patients treated with DMD were identified retrospectively. Patient and aneurysm characteristics, procedural findings, clinical outcomes and follow-up imaging results were evaluated. A total of 44 target aneurysms in 30 patients were treated with DMD. The mean age of the patients was 49.9 (range, 4–77 years). Four patients with five aneurysms presented with acute subarachnoid hemorrhage. The mean aneurysm size was 6.8 mm (range, 1.5–22 mm). In 29 (65.9%) aneurysms, adjunctive devices were used for endovascular treatment. The overall mortality rate was 3.3% and procedure-related mortality was 0%. Overall neurologic morbidity was 6.6% and none of the patients had a permanent sequela secondary to the procedure. The mean clinical follow-up period was 20.9 months (range, 3 days–46 months) and the mean DSA follow-up period was 10.9 months. A total of 37 (84.1%) aneurysms demonstrated total occlusion (Raymond–Roy [RR 1]); 3 (6.8%) aneurysms had a neck remnant or infundibular filling at the origin of the jailed side branch (RR 2), 4 (9.1%) aneurysms had residual aneurysm filling (RR 3). For those aneurysms treated with bare DMD, the total occlusion rate was 73.3% at a mean follow-up of 16.1 months. In this initial clinical single-center experience, DMD had a good safety profile and efficacy comparable with the currently used flow diverters.

## 1. Introduction

The introduction of flow diverter (FD) stents has changed the general landscape of interventional neuroradiology by enabling endovascular treatment of complex aneurysms such as fusiform, dissecting or blister aneurysms. These aneurysms were either not amenable to endovascular treatment or required deconstructive methods, for instance, parent artery occlusion, with relatively high complication rates. FD stents are speculated to exert a flow diversion effect mainly by two mechanisms: stagnation of the in-flow of the aneurysm and neointimal formation through the parent artery with resultant aneurysmal occlusion [1]. That said, the exact differentiation between an FD stent and a braided stent remains vague [2]. No clear-cut point exists to define where the effect of flow diversion definitely begins since it depends on several mechanical properties of devices including but not limited to metallic load, porosity and pore density.

The DERIVO^®^ mini Embolisation Device (DMD) (Acandis, Pforzheim, Germany) is a novel self-expandable flow diverter with a specific type of strut architecture. In this study, we present our single-center results of 30 patients with 44 intracranial aneurysms treated with DMD.

## 2. Materials and Methods

**Figure 1:**
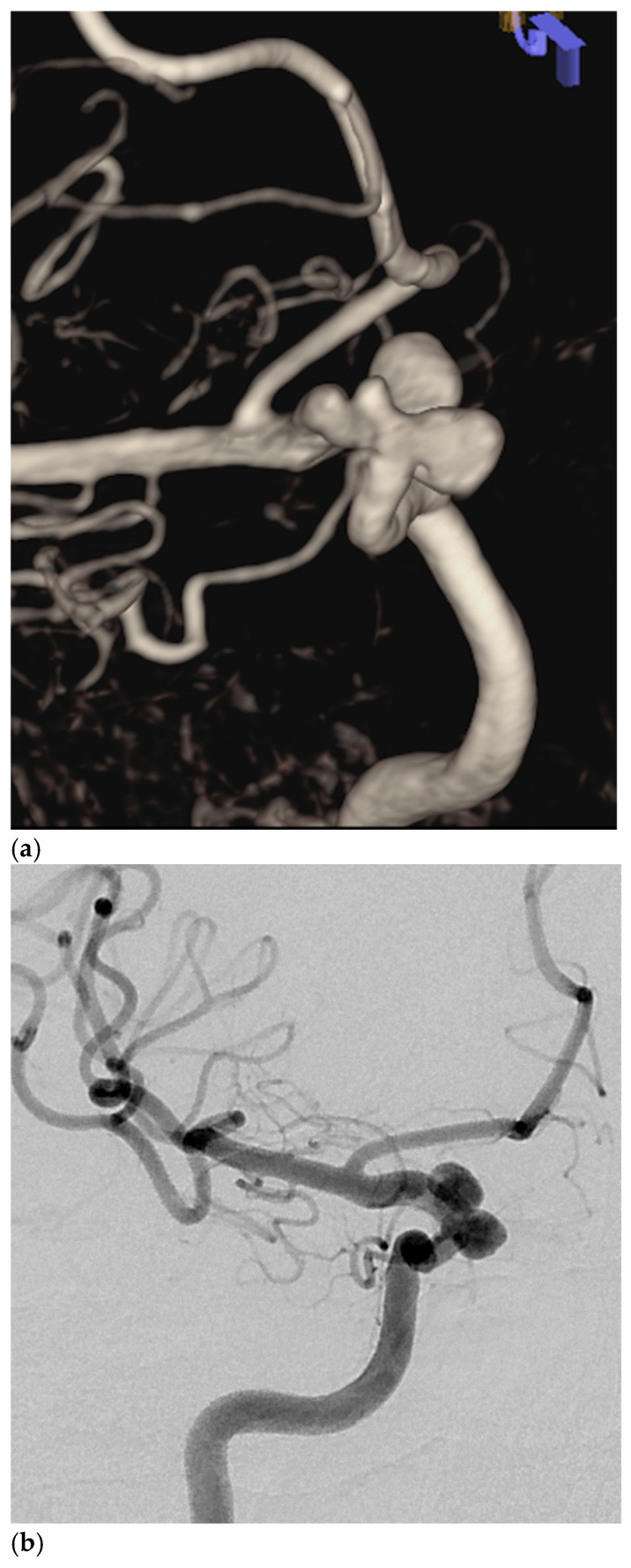

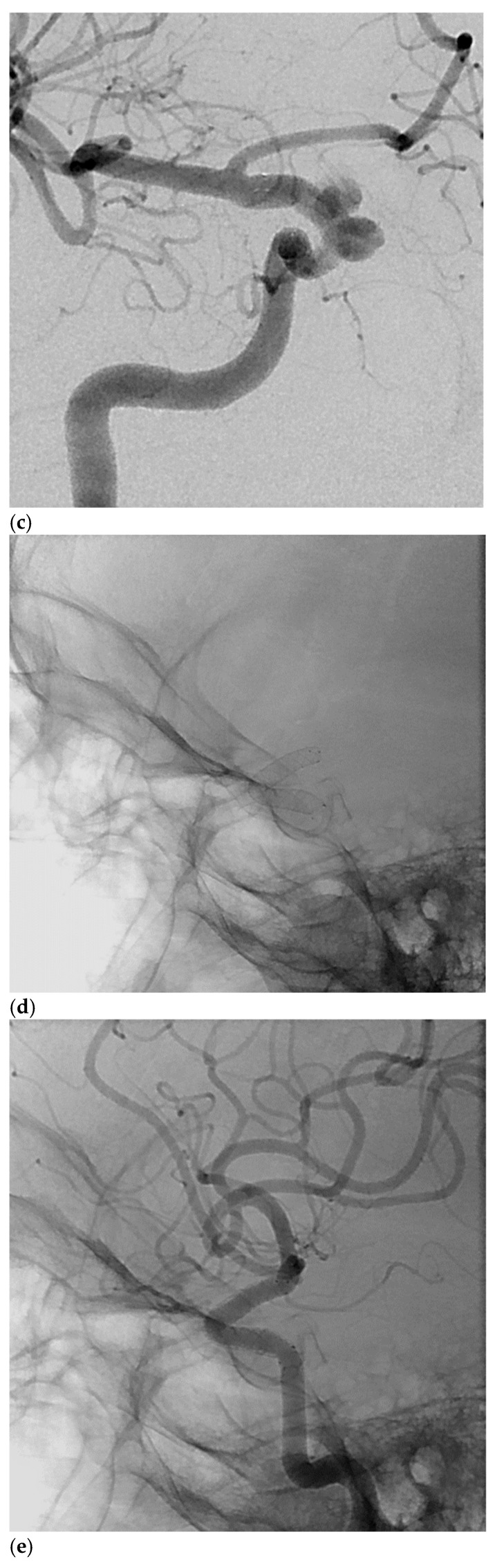

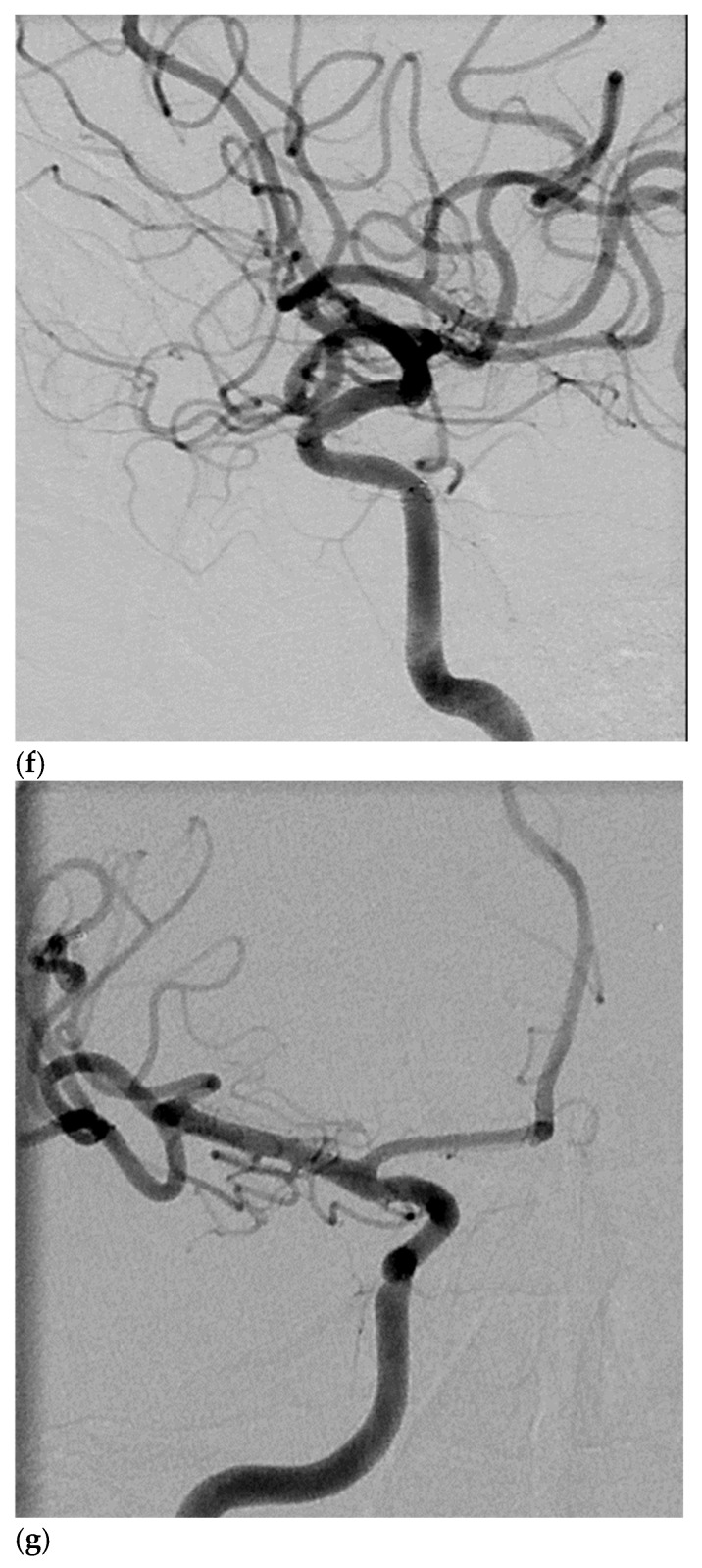
Volume rendered image of 3D rotational angiogram (**a**) and selective right carotid angiogram (**b**) demonstrate three tandem right ICA aneurysms. The aneurysms were treated with DMD only, which was seen on the selective carotid angiogram (**c**) obtained at the end of the procedure. The aneurysms were occluded, and the device was patent on native (**d**,**e**) and subtracted (**f**,**g**) images of the follow-up DSA.

The DERIVO^®^ mini Embolisation Device consists of a self-expanding braided nitinol device with 3 radiopaque markers each at the distal and proximal end. Both device ends are flared, and the distal end has closed loops. The nitinol composite wires have a platinum core for full visibility of the contour and each wire. Like DERIVO^®^, the DERIVO^®^ mini Embolization Device has the BlueXide^®^ surface finishing. The device is preloaded onto a transport wire in an introducer and is intended to be inserted using a microcatheter with an inner diameter of 0.021”. The DERIVO^®^ mini Embolization Device is available in diameters of 2.5, 3.0, and 3.5 mm and lengths of 15, 20 and 25 mm for the treatment of vessels from 1.5 mm (Figure 1a–g).

This retrospective study was approved by our institutional review board. We retrospectively evaluated all intracranial aneurysms treated in a single tertiary referral hospital. Patients treated with DMD—with or without adjunctive devices—were included in the study. Regarding the patient selection, we switch to 0.021-inch microcatheters to deploy DMD in cases of difficult and tortuous anatomy that precludes distal catheterization of the parent vessel with a 0.027-inch microcatheter.

For elective cases, our standard dual antiplatelet therapy was 75 mg of clopidogrel and 300 mg of acetyl salicylic acid (ASA) daily started at least 7 days before the procedure. In case of clopidogrel hyporesponse on point-of-care tests, clopidogrel was switched to 10 mg/day of prasugrel. For patients with acutely ruptured aneurysms, the patients were loaded intravenously with 8 mcg/kg of tirofiban during the procedure followed by continuous infusion of 0.1 mcg/kg/min for 12 h. After the procedure, the patients were loaded with 300 mg of clopidogrel.

All of the endovascular procedures were carried out under general anesthesia and systemic anticoagulation with a target activated clotting time between 200 and 250 ms. After catheterization of the target parent artery with an intermediate guiding catheter, the aneurysm neck was bypassed with a Prowler Select Plus (Codman, FL, USA) microcatheter. If coiling was planned in addition to flow diversion, the aneurysm sac was catheterized with a second microcatheter prior to the deployment of the flow diverter. The aneurysm occlusion status during the procedure and on follow-up imaging were noted down according to the Raymond–Roy (RR) occlusion classification. The patients were asked to undergo a very early noninvasive angiography around 6 to 8 weeks after the procedure and then a DSA follow-up 4 to 6 months after the procedure. The neurological status of the patients was evaluated during routine follow-up periods or by phone calls. The clinical results were noted down according to the modified Rankin scale (mRs). Patient demographics and aneurysm characteristics (location, size, shape), technical details related to the treatment procedures and perioperative clinical data were obtained from the hospital information system.

Statistical analyses were performed with the SPSS 25.0 (IBM Corp, Armonk, NY, USA) program. Descriptive statistics were given as percentages and means with standard deviation. Categorical variables were compared using the Chi-Square or Fisher’s Exact test. The statistical significance was set to *p* < 0.05.

## 3. Results

A total of 30 patients (11 males and 19 females) with 44 aneurysms were treated with DMD. The mean age of the patients was 49.9 ± 16.77 (range, 4–77 years). Two (6.7%) of them were pediatric patients; one of them had a posttraumatic dissecting right supraclinoid internal carotid artery (ICA) aneurysm, and the other had a dissecting right posterior cerebral artery (PCA) aneurysm.

The mean aneurysm size was 6.8 ± 4.57 mm (size range, 1.5–22 mm). The locations of the aneurysms were as follows: 17 (38.6%) middle cerebral artery (MCA), 14 (31.8%) ICA, 5 (11.4%) PCA, 4 (9.2%) anterior cerebral artery (ACA), 2 (4.5%) posterior communicating artery (Pcom) and 2 (4.5%) anterior communicating artery (Acom). A total of 20 (45.5%) aneurysms were sidewall aneurysms, and 24 (54.5%) aneurysms were bifurcation aneurysms. The aneurysm characteristics are presented in Table 1.

A total of 4 patients (13.3%) with 5 aneurysms (11.4% of the aneurysms) presented with acute subarachnoid hemorrhage (SAH). One of these patients had a diffuse subarachnoid hemorrhage secondary to bilateral, irregular shaped, flow-related Pcom aneurysms secondary to bilateral chronic ICA occlusions. The patient was in poor general condition owing to multiple accompanying medical problems including acute myocardial infarction, pulmonary edema and renal insufficiency. Preoperative WFNS grades for the ruptured aneurysms were as follows: 5 for the patient with bilateral true Pcom aneurysms, 1 for the patient with a blister-like ICA aneurysm, 4 for the patient with a tiny distal ACA aneurysm and 2 for the patient with a dissecting A1 segment aneurysm.

Three patients, two with Acom aneurysms and the other one with an MCA aneurysm, had a remote history of subarachnoid hemorrhage. Both Acom aneurysms had been treated with primary coiling in the acute phase in outside institutions.

In 15 (34.1%) aneurysms, adjunctive coiling was performed, whereas for 19 (43.2%) aneurysms, adjunctive stents were deployed (Table 2). In five (11.4%) of the aneurysms, the DMD was placed secondary to an unsatisfactory result obtained from stent-assisted coiling. In the remaining patients, stenting was performed mainly to pin the flow diverter device or to prevent possible fish-mouthing when the device was placed in arteries smaller than its approved diameter.

The patient with acute SAH and bilateral Pcom aneurysms passed away 4 days after the embolization secondary to the consequences of SAH and comorbidities; therefore, our overall mortality rate was 3.3%. We did not encounter any procedure-related mortality.

The patient with a ruptured tiny distal ACA aneurysm was emergently intubated upon presentation. After the treatment, his neurological status improved to mRS 3. We had two patients with periprocedural minor strokes. A patient with a remote history of SAH and multiple intracranial aneurysms had relatively slow flow in a jailed anterior temporal branch after the flow diversion. During awakening from general anesthesia, the patient developed negative pressure pulmonary edema and had to be re-intubated emergently secondary to hypoxia and profound hypotension. When the patient was extubated on postoperative day 1, upper extremity weakness was noted, which resolved over a week. In the second case, the patient experienced transient left hemiparesis on the day of the procedure. An immediate control DSA showed a patent device. The patient was managed conservatively, and her hemiparesis resolved spontaneously. All other patients were neurologically intact during periprocedural and postprocedural follow-up; therefore, our overall neurologic morbidity was 6.6%, and none of the patients had a permanent disabling stroke secondary to the procedure.

A patient with a large ICA ophthalmic segment aneurysm was treated with DMD. One month after the procedure, the patient developed headache and was found to have an acute small frontal intraparenchymal hematoma on noncontrast CT. Platelet function testing showed antiplatelet hyperresponse both on VerifyNow and Multiplate essays. Neurological examination remained normal, and the lobar hematoma did not expand on early imaging follow-up. After modification of the antiplatelet regimen to single antiplatelet therapy with 37.5 mg/day of clopidogrel, the patient was discharged uneventfully without a change in the neurological status.

All of the patients except for the patient with bilateral Pcom aneurysms had a follow-up DSA. This patient had a control CTA at 2 days after the procedure, which showed patent devices.

The mean clinical follow-up period was 20.9 ± 12.13 months (range, 3 days–46 months) and the mean last DSA follow-up period was 10.9 ± 9.8 months. The final DSA and final imaging follow-up results are summarized in Table 3. There was no statistically significant difference between aneurysms treated with bare DMD, stent-augmented DMD, and adjunctive coiling with respect to the occlusion rate (Table 3). When the last DSA follow-up results are taken into account, 33 (78.6%) aneurysms demonstrated total occlusion (RR 1), 4 (9.5%) aneurysms had a neck remnant (RR 2), and 5 (11.9%) aneurysms had residual aneurysm (RR 3). All aneurysms that were occluded on initial DSA remained occluded on subsequent MRA or CTA examinations. The final follow-up results were as follows: 37 (84.1%) aneurysms demonstrated total occlusion (RR 1), 3 (6.8%) aneurysms had a neck remnant or infundibular filling at the jailed side branch (RR 2), and 4 (9.1%) aneurysms had residual aneurysm filling (RR 3) at a mean follow-up of 20.9 months. When aneurysms treated with a bare DMD, that is, without adjunctive coiling and/or stenting, are considered, 11 (73.3%) aneurysms demonstrated total occlusion and 1 (6.7%) aneurysm had an RR class 2; 3 (20.0%) aneurysms showed residual filling at a mean follow-up of 16.1 months. When aneurysms treated with solely DMD +/− intracranial stents are evaluated, 23 (79.3%) aneurysms demonstrated total occlusion and 2 (6.9%) aneurysms had a RR class 2; 4 (13.8%) aneurysms showed residual filling at a mean follow-up of 15.9 months.

We did not encounter any technical challenge or failure during the deployment of the DMD through a 0.021-inch microcatheter. However, in one case, after deployment of the device, distal migration of the DMD occurred during the attempted microcatheterization of the device over a microwire, which necessitated deployment of a second DMD to provide optimal aneurysmal neck coverage. On follow-up imaging, all devices were patent with the exception of one case: a patient with an Acom aneurysm was treated with an Acclino (Acandis, Pforzheim, Germany) stent extending from the left ACA A1 to A2 segment and a DMD extending from the right ACA A1 to A2 segment with additional coiling. The proximal part of the DMD at the right ACA A1 segment was occluded on 5-month follow-up DSA; the right A2 segment was filling from left A1 via the anterior communicating artery. Two patients needed retreatment, one with a blister-like ICA aneurysm for persistent filling on 1-month follow-up imaging. Another patient with an MCA aneurysm was treated with Enterprise (Codman, Raynham, MA, USA) stenting in order to address the fish-mouthing of the device.

## 4. Discussion

The factors related to aneurysm occlusion after placement of a flow diverter are still a matter of debate [3]. As a matter of fact, a level of flow diversion that distinguishes a braided stent from a flow diverter does not currently exist. Given the results of computational fluid dynamics (CFD) studies [4] and the high rate of aneurysm occlusion at 12 months, the porosities and pore densities of the current flow diverters set a standard for flow-diverting devices [5].

On the other hand, it is still not possible to denote a device as a “flow diverter” based on only porosity or metal coverage. It is known that a clear-cut correlation between porosity and efficacy does not exist [4]. Especially for idealized (i.e., non-bifurcation) aneurysms created for CFD modelling, increased efficacy of flow diversion with decreased porosity remains controversial [6]. These CFD findings are also supported by animal models, in which lowered porosity/increased pore density (by increasing the braid number from 36 to 48) did not directly translate into increased aneurysm occlusion scores or neointimal coverage [7]. That is to say, there may not be a ‘benchmark’ minimal porosity—or even pore density—that will unequivocally lead to the occlusion of all aneurysms [5].

Relatively new data suggest that FD porosities around 70% may be sufficient to initiate thrombus formation within the aneurysm [4,8]. Lieber et al. put forward that a device with metallic wall coverage of as low as 24% (porosity of 76%) can potentially act as a flow diverter [9]. Due to the fact that the actual metallic coverage rate of FDs is in fact lower than the nominal value of 30–35% [10], a cut-off value for actual wall coverage at 24% may indeed be plausible for flow diversion. Newer data propose that even lower percentages of wall coverage may suffice for flow diversion. Jou et al. [10] pointed out that a homogenous stent with 80% porosity will decrease the intraaneurysmal flow enough to reach values well below 0.025 m/s, which is a value proposed to be critical for thrombus induction in the aneurysm [1,4]. Similarly, simulation studies have revealed that flow within an aneurysm was reduced substantially at around 86% porosity [4], and this value is practically the lower limit of porosity for braided stents, potentially representing the maximal porosity that can be used to denote a stent as a flow-diverting device.

That being said, a metallic coverage ratio higher than flow diverters may be attainable with braided intracranial stents after device compaction [11]; even then, the braided stents may not be able to provide the ‘standard’ level of flow diversion due to their lower pore density. A braided stent can be forcefully shortened to decrease the pore size to 424 μm [12], a value below the pore size of 500 × 500 microns, which is known to be sufficient for intraaneurysmal stasis. CFD studies suggest that even under such conditions, the stent will not lower intraaneurysmal flow velocities as much as a flow diverter despite attaining similar porosity values (about 65%) [11,12]. Hence, braided stents remain significantly different from flow diverters in terms of their capacity for flow diversion [13]. This proposition remained valid in clinical series. For instance, in a clinical series by Koch et al., a braided stent with a metallic wall coverage of up to 28% (LVIS Blue, Microvention, CA, USA) was proposed as a stand-alone flow diverter [14]. However, in this series, 2 out of 10 patients were treated with multiple devices yet the 6-month occlusion rate remained only at 40%. The discrepancy in the end result with the expected result is a consequence of a multitude of device-related parameters including the braiding angle, braiding pattern, wire diameter, mechanical deformation of the device, wall apposition and uniformity of the pore structure/device and availability of appropriate sizes [3,5,7,15]. In other words, stent design may be a critical determinant of flow diversion beyond wall coverage and pore density [16], but our current knowledge is far from predicting flow diversion based on stent architecture.

Consequently, a braided device can claim to be a flow diverter only when its clinical and angiographic outcomes are concordant with those of the currently accepted flow diverters. As expected, the occlusion rates of our patients were high when adjunctive devices were used. The safety profile was acceptable with no procedure-related mortality or permanent neurologic morbidity, suggesting that the specific device structure did not have an adverse impact on the deliverability or deployment of the device during the procedure or on the rate of device stenosis/occlusion (2/30%). As an additional technical note, although we did not encounter technical failure during navigation and deployment of the DMD in our cohort, due to the relatively low expansile force of the device, slight oversizing relative to the diameter of the parent vessel should be considered in order to provide optimal wall apposition.

Apart from the DERIVO^®^ mini Embolisation Device, there are other flow diverter devices compatible with 0.021-inch microcatheters including the FRED Jr (Microvention, Tustin, CA, USA), Pipeline Vantage (Medtronic, Dublin, Ireland), and p48MW/p64MW (Phenox, Bochum, Germany) devices. Vivanco-Suarez et al. conducted a meta-analysis comprising a total of 156 aneurysms treated with a p48MW device with a complete occlusion rate of 67% at a follow-up period of 2–13.1 months [17]. In a meta-analysis by El Naamani et al., the complete occlusion rate for the FRED Jr device was 69.9% in 244 distal aneurysms located on small parent vessels [18]. The results of the previous major studies dealing with the aforementioned FD stents (FD stents compatible with 0.017- and 0.021-inch microcatheters) are summarized in Table 4. The efficacy of the DMD, which has a range of porosity below the braided stents, a pore density in the range of the earlier Silk device [19,20], and a braid number that is similar to that of the initial Tubridge device [21], achieved a total occlusion rate of 73.3% for the “bare DMD” group. The last DSA follow-up occlusion rate in this subgroup of our patients is somewhat lower than the 6-month occlusion rates obtained with similar lower profile devices mentioned above [22]. This finding suggests that the pore density of the DMD is somewhat lower than these devices but there may be a late “catch up” for total aneurysmal occlusion. On the other hand, a higher porosity as compared to devices with a similar profile may be beneficial in terms of reducing the risk of side-branch occlusion for aneurysms that are adjacent to important side branches such as anterior choroidal aneurysms, ophthalmic aneurysms and blister-like aneurysms (for both anterior choroidal and anterior cerebral arteries) and also for sidewall aneurysms located on perforator-rich arterial segments.

Previous studies in the literature dealing with cost-effectiveness consistently demonstrated that flow diversion remains a cheaper endovascular treatment method, which becomes more remarkable for larger aneurysms requiring higher number of coils for occlusion [23,24]. Although these studies generally included the patients treated with a Pipeline Embolization Device at the FD arm, the conclusions reached by these studies seem generalizable to other FD stents in the market as well as to DMD.

In spite of the encouraging results, the current study has several limitations: first, being a single-center retrospective study, it is inherently subjected to selection bias, which may be further augmented by the patient selection criteria. Second, although the results of previous studies conducted with similar FD devices are summarized in Table 4, the lack of a control group precludes the comparative evaluation of the findings of the current study. Third, our study, comprising 30 patients with 44 aneurysms, has a small sample size; therefore, our results need be validated further with larger studies. Fourth, our follow-up period of 20.9 months is relatively acceptable when compared to the existing literature; however, studies with longer clinical and imaging follow-up periods allow more robust conclusions to be drawn. On the other hand, it outlines the utility of the device in a heterogenous group of patients reflecting real-life settings.

In conclusion, in this initial series, the efficacy and safety of the DERIVO^®^ mini Embolisation Device were on a par with the currently used larger profile flow diverters. Further utilization of the DERIVO^®^ mini Embolisation Device, preferably evaluated in multicenter, prospective, randomized controlled studies with longer follow-up results, will enable better comparison of the device with similar devices.

**Table 4:** The previous literature on FD stents similar to DMD.

	FD Stent	Patient/ Aneurysm Number (n)	Mean Age (years)	Mean Aneurysm Size (mm)	Mean Follow-Up Period (months)	Aneurysm Occlusion Rate (%)	Permanent Neurological Morbidity/Mortality
Bhogal et al. [25]	p48MW	25/25	55.0	4.7	13.1	75.0	None/ One patient unrelated to the treatment
Schob et al. [26]	p48MW/ p48MW HPC	32/32	59.0	N/A	N/A	N/A	None/None
Hellstern et al. [27]	p64MW HPC	102/132	58.1	4.8	N/A	83.8	None/None
Petrov et al. [28]	p64MW HPC	29/46	57.0	3.7	6.8	85.0	None/None
Bhogal et al. [29]	p48MW HPC/ p64MW HPC	24/30	48.2	8.2	6.1	64.7	None/None
Winters et al. [30]	p64MW HPC	32/33	57.0	N/A	5.9	65.2	None/None
Pumar et al. [31]	Silk Vista	25/27	58.0	7.0	3.4	77.7	None/None
Martínez-Galdámez et al. [32]	Silk Vista	60/60	N/A	N/A	N/A	8.0	One patient related to pontine stroke/ None
Da Ros et al. [33]	Silk Vista	48/48	N/A	10.1	8.1	76.0	One patient related to subarachnoid hemorrhage/ One patient unrelated to the treatment
Jesser et al. [34]	FRED Jr	150/159	55.0	6.7	24.0	83.0	Two patients (1%)/ None
Sayin et al. [35]	FRED Jr	25/31	48.8	5.9	21.0	95.2	One patient related to delayed intraparenchymal hemorrhage/ Two patients unrelated to the treatment
Current study	DMD	30/44	49.9	6.8	20.9	84.1	One patient related to subarachnoid hemorrhage/ One patient unrelated to the treatment

DMD: DERIVO^®^ mini Embolisation Device, FRED: Flow Redirection Endoluminal Device, HPC: Hydrophilic Coating.

## Figures and Tables

**Table 1:** Aneurysm characteristics.

Characteristics	Mean or Number (Percentage)
**Aneurysm location**	
Middle cerebral artery	17 (38.6)
Internal carotid artery	14 (31.8)
Posterior cerebral artery	5 (11.4)
Anterior cerebral artery	4 (9.2)
Posterior communicating artery	2 (4.5)
Anterior communicating artery	2 (4.5)
Mean aneurysm size ± standard deviation (mm)	6.8 ± 4.57
Number of acute ruptured aneurysms	5 (11.4)
Number of aneurysms treated with bare DMD	15 (34.1)
Number of aneurysms treated with bare or stent-augmented DMD	29 (65.9)
Number of aneurysms treated with adjunctive coiling	15 (34.1)

DMD: DERIVO^®^ mini Embolisation Device.

**Table 2:** Type of treatment, n (%).

	Bare DMD	Stent-Augmented DMD
Without coiling	15 (34.1)	14 (31.8)
With coiling	10 (22.7)	5 (11.4)

DMD: DERIVO^®^ mini Embolisation Device.

**Table 3:** Post-treatment follow-up results.

	RR Class 1	RR Class 2	RR Class 3	*p* Value
**Last DSA follow-up results, n (%)**				0.214
Overall	33 (78.6)	4 (9.5)	5 (11.9)	
Aneurysms treated with bare DMD (without coiling)	9 (69.2)	1 (7.7)	3 (23.1)	
Aneurysms treated with stent-augmented DMD (without coiling)	10 (71.4)	3 (21.4)	1 (7.1)	
Aneurysms treated with adjunctive coiling	14 (93.3)	-	1 (6.7)	
**Last imaging follow-up results, n (%)**				0.493
Overall	37 (84.1)	3 (6.8)	4 (9.1)	
Aneurysms treated with bare DMD (without coiling)	11 (73.3)	1 (6.7)	3 (20.0)	
Aneurysms treated with stent-augmented DMD (without coiling)	12 (85.7)	1 (7.1)	1 (7.1)	
Aneurysms treated with adjunctive coiling	14 (93.3)	1 (6.7)	-	

DMD: DERIVO^®^ mini Embolisation Device, RR: Raymond–Roy.

## Data Availability

The raw data supporting the conclusions of this article will be made available by the corresponding author on reasonable request. The data are not publicly available due to containing information that could compromise the privacy of the patients.
